# Public health round-up

**DOI:** 10.2471/BLT.26.010626

**Published:** 2026-06-01

**Narrowing the global vaccination gap**

The Big Catch-Up, a global campaign that started in 2023 to reverse global declines in childhood vaccination has reached 18.3 million children under five years of age across 36 countries, delivering over 100 million vaccine doses. Around 12.3 million were “zero-dose children” who had not previously received any vaccines and 15 million who had never received a measles vaccine. With implementation ending in March 2026, the Big Catch-Up is expected to meet its goal of reaching at least 21 million children.

**Figure:**
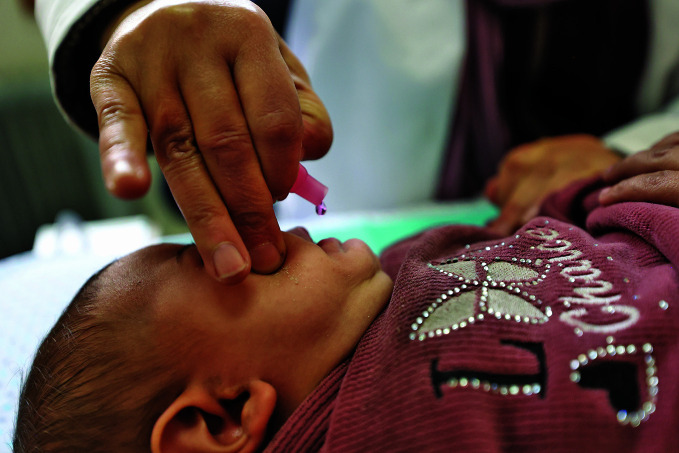
WHO

## Hantavirus outbreak

On 2 May 2026, the World Health Organization (WHO) received notification from the national focal point of the *International health regulations* (IHR) in the United Kingdom of Great Britain and Northern Ireland regarding a cluster of severe acute respiratory illness aboard the Dutch-flagged cruise ship MV Hondius.

As of 13 May, a total of 11 cases (eight confirmed, one inconclusive and two probable cases), including three deaths (two confirmed and one probable), have been reported. 

Five of the cases have been confirmed as Andes virus, the only hantavirus known to allow limited human‑to‑human transmission through close, prolonged contact. WHO Director-General Tedros Adhanom Ghebreyesus described the situation as serious, but stressed that WHO currently assesses the overall public health risk as low. He cautioned, however, that more cases may emerge given the virus’s incubation period.

WHO is coordinating with several countries under the *International health regulations*, highlighting the importance of global cooperation in responding to cross‑border health threats. 

Since being notified, WHO has deployed an expert to the ship to conduct medical assessments and gather information on potential exposure risks. The Organization has also arranged the shipment of 2500 diagnostic kits from Argentina to laboratories in five countries to expand testing capacity. 

https://bit.ly/3PCPbXy

## World health statistics 2026

The *World health statistics 2026* report, published on 13 May 2026, warns that global progress on health is slowing, uneven, and in some areas reversing, leaving the world off track to meet any health‑related sustainable development goals by 2030. The report highlights meaningful gains over the past decade, such as new HIV infections down 40%, major declines in tobacco and alcohol use, and a 36% drop in people needing interventions for neglected tropical diseases. Access to essential services has also expanded, with billions gaining improved water, sanitation, hygiene and clean cooking solutions. Some regions, including Africa and South‑East Asia, have achieved faster‑than‑global progress against HIV, tuberculosis and malaria.

Yet major challenges persist. Malaria incidence has risen 8.5% since 2015, anaemia affects nearly one third of women of reproductive age, and childhood overweight continues to grow. Violence against women remains widespread and trends on maternal mortality, child mortality and noncommunicable diseases remain off track. Progress towards universal health coverage has stalled, with one quarter of the world facing financial hardship from health costs. “These trends reflect too many deaths that could have been avoided,” said Yukiko Nakatani, WHO assistant director-general for health systems, access and data. “With rising environmental risks, health emergencies, and a worsening health financing crisis, we must act urgently – strengthening primary health care, investing in prevention, and securing sustainable financing to build resilient health systems and get back on track.” 

https://bit.ly/48Y8HnQ

## Curbing salt intake

WHO has released a new edition of its SHAKE technical package to help countries reduce dangerously high salt consumption, a major driver of hypertension and cardiovascular disease. Global sodium intake remains more than double WHO’s recommended limit, contributing to an estimated 1.7 million deaths in 2023. Despite longstanding public health advice, most people live in food environments dominated by processed and packaged foods high in sodium, leaving the world far off track to meet the 2030 target of a 30% reduction in salt intake.

The updated SHAKE package provides stronger, more actionable guidance for governments, emphasizing mandatory, government‑led policies. It offers a structured framework: Surveillance, Harness industry, Adopt standards, Knowledge and Environment, to support coordinated national action. “Excess salt consumption remains among the top preventable drivers of death globally, and implementing mandatory policies to reduce sodium intake is one of the most cost-effective actions countries can take to protect people from cardiovascular disease,” said Luz Maria De Regil, director of WHO’s Department of Nutrition and Food Safety.

The package outlines proven measures including food reformulation, front‑of‑pack labelling, healthier food procurement, marketing restrictions, taxation of unhealthy foods, public education campaigns and lower‑sodium salt substitutes. WHO urges governments to adopt bold national targets and transform food environments to make reducing salt intake achievable. 

https://bit.ly/4tSyNjX

## Health care in armed conflicts 

On the tenth anniversary of United Nations (UN) Security Council resolution 2286 on health care in armed conflicts, adopted at its 7685th meeting on 3 May 2016, the heads of the International Committee of the Red Cross, WHO and Médecins Sans Frontières issued a joint warning that violence against health care in armed conflict has worsened, not improved. They stated that the day marks not an achievement, but a failure, as attacks on hospitals, ambulances, medical staff and patients continue unabated across multiple crises. 

The organizations report that when health care is targeted, preventable deaths rise, women give birth without adequate care, and entire communities lose access to lifesaving services, signalling a broader breakdown of respect for the laws of war.

The three leaders urged states and all parties to conflict to uphold their obligations under international humanitarian law, stressing that states must not only comply themselves but use their influence to ensure others do the same. They called for concrete action to implement resolution 2286, integrate protection of health care into military doctrine, strengthen domestic laws, allocate adequate resources, influence partners in conflict, investigate attacks transparently, and report regularly on progress.

They emphasized that the continued targeting of health care reflects not a failure of the law but a failure of political will, urging world leaders to act decisively. Health care, they stressed, must never be a casualty of war. 

https://bit.ly/4uhLx4E

## Strengthening substance control 

The UN Commission on Narcotic Drugs has placed three new psychoactive substances under international control, following scientific recommendations from the WHO Expert Committee on Drug Dependence. 

The substances, two synthetic opioids, N‑pyrrolidino isotonitazene and N‑desethyl etonitazene, and the synthetic cannabinoid MDMB‑FUBINACA, have no recognized medical use and have been linked to fatal overdoses and severe poisonings. Countries must now apply control measures under the international drug conventions, strengthening the global response to emerging synthetic drugs often found in falsified tablets, herbal mixtures and vaping liquids.

The Commission also reviewed WHO’s first comprehensive assessment of coca leaf in more than 30 years. WHO recommended maintaining coca leaf in Schedule I of the 1961 Single Convention on Narcotic Drugs, a position the commission confirmed. The review acknowledged that traditional coca chewing and coca tea pose limited public health risks and hold cultural significance for Indigenous Peoples, but warned of the rapidly expanding cocaine market and the ease of converting coca leaf into cocaine. Current scheduling remains necessary to mitigate risks while allowing traditional and legally permitted uses.

https://bit.ly/48Yax8e

## Understanding skin-lightening practices

WHO has released a behavioural insights toolkit to help countries understand and address the drivers of harmful skin‑lightening practices, many of which involve mercury‑containing products that pose serious health and environmental risks. 

The toolkit, developed through a multicountry project, supports governments in analysing the psychological, social and cultural factors behind strong consumer demand in a global market projected to reach 16.4 billion United States dollars by 2032. 

Drawing on pilot projects in Gabon, Jamaica and Sri Lanka, it includes tools such as user‑journey mapping to identify key intervention points. The toolkit aligns with global efforts under the Minamata Convention to eliminate mercury‑containing cosmetics.

https://bit.ly/4twJUi4

## First malaria treatment for newborns and infants

WHO has prequalified the first malaria treatment designed specifically for newborns and infants weighing between two and five kilograms. The new artemether‑lumefantrine formulation meets international standards for quality, safety and efficacy, enabling public procurement and closing a long‑standing treatment gap for the roughly 30 million babies born each year in malaria‑endemic regions. Until now, the youngest patients have relied on medicines intended for older children, increasing risks of dosing errors and toxicity.

https://bit.ly/4fkEoM2

**Cover photo**

Houses submerged by floodwaters in Chiaquelane, Chókwè District, Mozambique, February 2026.

**Figure:**
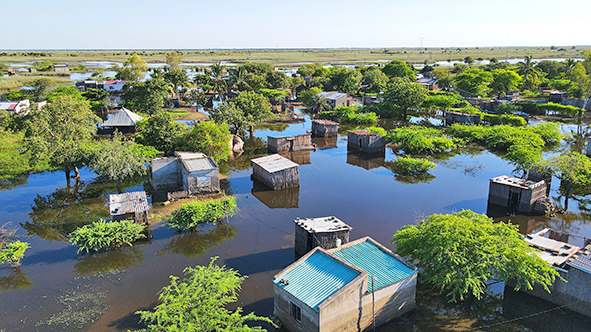
WHO/Júlio Dengucho

**Looking ahead**

7 June. World Food Safety Day. https://bit.ly/42yrC5a

22–24 June. 3rd Global Convening of the Global Initiative on Digital Health. Geneva, Switzerland. https://bit.ly/4wtsHc0

25 June. Alliance for Transformative Action on Climate Change and Health (ATACH) High-level meeting. Paris, France. https://bit.ly/4uJXmQy

